# The “Science” of Medication

**Published:** 1884-07

**Authors:** C. S. Carr


### THE "SCIENCE" OF MEDICATION.

BY C. S. CARR, M. D.

While it will be reluctantly admitted that "doctors" are utterly important to cure all diseases termed chronic, hereditary or organic, the majority of mankind are not prepared to believe that all diseases are quite beyond the ken or the cure of even "the regular physician." Who is there that has not had a relative or friend suddenly stricken down with some acute malady, frightening the whole household into a frantic desire to do something immediately, when the doctor is summoned at breakneck speed, who arrives just in time to save the unfortunate victim from speedy dissolution.

Then follow the long weeks of watching and waiting, hoping and fearing, the monotony of death-like stillness pervading the house, unbroken save by the frequent and regular visits of the doctor. The crisis comes at last, the sufferer passes it in safety, and is soon restored to health.

The doctor has "brought his patient through," so they all exclaim in unfeigned gratitude. It is not to be expected that the witnesses to such a common experience of this life would be easily persuaded that the doctor has done absolutely nothing to the cure of his patient. They cannot so easily forget the profound impression produced by the pompous manner, the knowing looks, the unintelligible words of the disciple of Esculapius, who for the pure love of "science" has been so attentive to the welfare of the saved one. It does not seem possible that such swagger, such gorgeous pomposity, can indicate aught else but fathomless wisdom and most penetrating intellect.

It would be difficult to convince the average spectator that the deluge of drugs with which they have so vigorously assailed the patient from the first to the last were not the very elixir of life to the afflicted one. Then those dark hours when the crisis had been reached, when it seemed to all interested that the life of the patient, hung by a single thread, the doctor pacing up and down the room with brows knit, as if in deep study, evidently evolving with great mental parturition some long lost idea which was to turn the balances of life in favor of the sufferer, it would be hard, very hard, to convince one of that grateful household (the patient excepted) the whole transaction from beginning to end was a farce, a childish farce. But substitute in the place of the non-recognized medical treatment the recognized treatment of thirty years ago, and the whole medical fraternity, without reference to school or kind, would unanimously join me in pronouncing it a serious farce—yes, worse than a farce, absolutely murderous. What will the medical profession of thirty years hence say of our treatment to-day? The medical treatment that George Washington received during his last illness was considered at that time just as scientific as the treatment that the late lamented James A. Garfield received. Now, in reference to the principal acute diseases which are especially dreaded by the people, and which constitute the principal opportunities for the display of "great medical skill," probably four of the most common ones will serve to illustrate the amount of knowledge that the profession is in possession of in this most enlightened age, which is supposed to eminently qualify them to successfully combat these diseases. We will select typhoid fever, diphtheria, scarlet fever and malaria. It will be conceded by all, no doubt, that a physician, in order to intelligently treat a disease, ought to know something of the nature of the cause. Every thoughtful person would join, without a moment's hesitation, in denouncing a physician as entirely incompetent to treat a disease until he understood the nature of the disease. But to call a disease typhoid fever throws no light on its nature. The word typhoid means "like unto typhus," and typhus means stupor, so we have very feeble illumination of the subject to know simply that it is a fever resembling stupor.

Diphtheria, like typhoid, comes from the Greek, and means in English "a membrane," which by itself would furnish only a meager amount of information to enable any one to successfully treat it. Scarlatina (scarlet fever) is derived from the Italian, and signifies "deep red," while malaria in plain English is "bad air." It must be quite apparent that we shall be compelled to find a little more instruction than is suggested by these names before we can be said to have any knowledge about the diseases. Now, let us consult the medical works which furnish to the physicians regular or irregular alike all the deep learning they are supposed to be in possession of, and which comprises their entire qualifications to combat these diseases.

The quotations need not be lengthy since so many medical writers use no circumlocution in reference to their entire ignorance as to true cause of these as well as all other diseases. It is supposed by some medical authors, perhaps by the majority, that the cause of either of the above-mentioned diseases is the presence in the system of vegetable organisms called spores, bacteria, or disease germs. It is also supposed that the difference manifested by the diseases is due to a difference in the nature of the organisms. We say supposed, for the whole theory is a chain of conjectures, based upon very uncertain and meager observations. In "Ziemssen's Cyclopedia of Medicine," vol. 2, p. 38, in reference to the cause of measles, we find the following: "The nature of the contagious principle of measles is still entirely unknown." No language can be stronger, it simply amounts to a confession of absolute ignorance of the cause of measles. Now let us see how much is known of the cause of malaria. As before, we quote from the standard medical authority of America and Europe, "Ziemssen's Cyclopedia." On p. 584, 585, vol. 2, is found the following: "The necessary conclusion from all this is, that the telluric and atmospheric influences referred to above are not sufficient to account for the origin of malaria, and that there must be additional causes at work thus far unknown to us." "Some think that they find these causes in the exhalations from living plants, in the ethereal oils, inasmuch as the prevalence of malaria corresponds not with the decay, but with highest development of the plants." "Others attribute the poison to subterranean exhalations, to the gaseous effluvia from volcanic soil; still others deny any specific cause, or, again, believe it to consist in an accumulation or modification of the electricity of the earth and air."

"None of these views have as yet been established by sufficient proof; and even the supposed microscopic vegetable organisms, of low grade, the spores and algæ, with regard to the influence of which in producing malaria so much has been said, lack as yet all practical conformation." That is to say, nothing whatever is known concerning the cause of malaria, positively nothing. Its origin, its nature, its modus operandi, is as securely hidden from the scientific doctor of the present, as it was from the savage doctor of the past. We trust the brevity of the quotations cited will not lead any one to infer that admissions similar to the above are exceptional in medical literature, that we have ransacked medical works for the purpose of finding a few inadvertant admissions of careless writers, for such is not the case, but on the contrary the above quotations represent fairly the unanimous testimony of all medical works." As to diphtheria the following will suffice to indicate the amount of information the medical fraternity are in possession of up to date:

The Medical Record of March 29, 1884, p. 346, has this quite characteristic contribution to medical science, communicated by T. J. Hutton, M. D., formerly resident physician of Long Island College Hospital, etc., etc.

"Few boast when diphtheria is discussed. Both lay and professional refer to it with qualms of the solar plexus. Some of our best men have again been consulted on this subject, but have nothing new to offer; they are silent as to the cause; they give no statistics of results; they differ as to the indications; and they distinctly enumerate forty-three different remedies that might be of use in an ordinary case of diphtheria. Then, if these do not suffice symtomatics, ruberants, stimulants, febrifuges, externally, internally or hypodermically, may be drawn on. If there be irony in these facts it is not mine, and as to these men we hold them in the highest esteem; they are among the ornaments of our profession. Then, too, a glance at the mortality lists tells how much we do not know about diphtheria—the sepulchres make all the ground uneven. Can nothing be done? else of what use is the healing art? Why should it not fitly fall into ridicule, since when assistance is most needed, as in this malady it can render little or none?" No text book even pretends to teach anything that throws any light on this dark subject. Profound ignorance envelops the whole subject of diphtheria, and yet doctors thrive in consequence of their supposed knowledge of this terrible disease. No more is known of the cause of typhoid fever than of the others. All authors alike deliberately confess their entire want of information. In Bartholow's "Practice of Medicine," p. 741, fifth edition, we learn that "typhoid fever" owes its origin to a peculiar poison, whose source and nature have thus far eluded investigation." The above quoted statement, or its exact equivalent, is to be found in every text book upon the subject. Exactly as much is known of the topography of the other side of the moon as is known of the cause of either of the above diseases. There can be no one who knows less of the real nature of these diseases than the regular physician."

The charlatan differs from the scientific doctor not in knowing less, but only that he is less positive that he knows nothing. To know nothing of disease without being aware of it is to be a quack, but to know that nothing is known about disease is to be an educated physician. The educated physician knows more than the quack, simply because he knows that he knows nothing, while the quack ignorantly supposes he really knows something. The only rational theory that was ever projected in reference to the cause of all epidemic or endemic diseases is the germ theory. Take away the germ theory, and not even a conjecture would be left. The germ theory never rose above a vague probability, but according to the latest research it has ceased to be even a probability. That germs or organisms has some relation to some diseases is a strong presumption, but that they stand in relation to disease as a cause is highly improbable. That germs are only one of the effects of disease is held by some, that they bear no relation to disease is held by others, still others cling with a death-grip to the guess that they are the cause of disease, which is not a strange thing, since it is the only clue to the cause of disease ever imagined. The present position of the germ theory as to the cause of disease is fully set forth in an editorial in the Philadelphia Medical and Surgical Reporter, quoted by the Microscope of May, 1884, vol. 4, p. 114. "The rapid strides which pathological histology is making are gradually overtaking the elusive cause of disease." Hunted down to the lowest forms of animal and vegetable organisms, it may now be said to have been driven out of them and into a smaller field, in which we are close upon its tracks. "Not that the relations of germs, bacteria and bacille are no longer of importance; they are of great importance, but they are no longer directly causal, only accessory to the true causes, as these are now understood." . . . . . "It has long been suspected that the bacille, etc., are rather the carriers of disease than its direct producers, and the inquiry has been directed toward what it is they carry." Is it a chemical poison or what? . . . . . . The immediate future of the study of the causes of disease lies in the separation of the coincident phenomena from the real cause. . . . . . "The field thus opened is even a more difficult one than that of microscopical life; it belongs to pathological chemistry, and the methods for its successful exploration have scarcely as yet been discovered." In other words, they have just discovered that former discoveries were scarcely worth the discovering, and they have further discovered that they must discover some other discovery before they will be able to discover the real cause of disease. Real cause, indeed! what other cause is imaginable but the real cause? We are led to suppose that there is one real cause of a disease, and a dozen or so unreal causes lying about loose as decoys.

If we may be allowed to assume that a doctor with no acquaintance with the cause of a disease is not able to treat it intelligently, then it follows as a matter of necessity that no epidemic or endemic disease has ever been as yet intelligently treated. But it may be urged that while the physician is not able to treat the disease specially, yet he may by proper medication, mitigate somewhat the sufferings of the victim of disease. He is able to produce sleep, quiet pain, allay restlessness, etc., etc. And it must be confessed that it does read well in books, but follow us to the bedside of the patient and witness the supposed beneficient effect of palliative medication, and it will be painfully apparent that drugs may produce stupor, but not sleep. They are able to paralyze sensation, but not to remove pain; stultify they may, but never can they give rest. The quiet produced by drugs is simply the insensibility of transient paralysis. The stimulation of drugs is but the momentary irritation of some nerve centre, to be followed by depression exactly corresponding in amount and duration, to the stimulation received. In a normal condition of the nervous system the nerves are able to notify the sensibilities of any derangement within the territory of their distribution; all palliative medication is but an attempt to render any given nerve unable to enter its protest to the sensibilities while it leaves the disease entirely untouched. The disease in its real nature being as much a mystery to the physician as to the patient, leaves medicine entirely impotent to mitigate or cure. The science of medicine has rendered humanity a noble service, for it has taught the people that epidemic and contagious diseases are somehow or other attributable to filth and uncleanliness, and thereby the number of epidemics as well as their severity have been greatly diminished. But in spite of all prophylactic measures a disease breaks out among the people, the physician is utterly unable to cure or palliate, as with the well, so it is with the sick, rest can only be obtained by normal sleep and strength, by nutrition, and medicine can produce neither the one nor the other. Cleanliness and moderate industry in health, good nursing and rest in disease, these are the nucleus of truth around which cluster the thousand uncertainties which constitute the "science" of medicine.

